# *In vitro* Ploidy Manipulation for Crop Improvement

**DOI:** 10.3389/fpls.2020.00722

**Published:** 2020-06-03

**Authors:** Darren H. Touchell, Irene E. Palmer, Thomas G. Ranney

**Affiliations:** Mountain Crop Improvement Lab, Department of Horticultural Science, Mountain Horticultural Crops Research and Extension Center, North Carolina State University, Mills River, NC, United States

**Keywords:** chromosome doubling, *in vitro* regeneration, mitotic inhibitor, plant breeding, polyploidy, whole genome duplication

## Abstract

*In vitro* regeneration systems provide a powerful tool for manipulating ploidy to facilitate breeding and development of new crops. Polyploid induction can expand breeding opportunities, assist with the development of seedless triploid cultivars, enhance ornamental characteristics and environmental tolerances, increase biomass and restore fertility in wide hybrids. *In vitro* ploidy manipulation is commonly induced using antimitotic agents such as colchicine, oryzalin and trifluralin, while many other antimitotic agents have been relatively unexplored. Successful induction requires a synergistic pairing of efficient penetration of the antimitotic agent and may be dependent the length of exposure and concentrations of antimitotic agents, tissue types, and interactions with basal media and plant growth regulators. *In vitro* conditions vary among taxa and individual genera, species, and cultivars, often requiring unique treatments to maximize polyploid induction. In some taxa, the induction of polyploidy influences *in vitro* growth, development, and root formation. Here we provide an overview of mitotic inhibitors and their application for *in vitro* ploidy manipulation for plant breeding and crop improvement.

## Introduction

Polyploidy, the condition of having more than two sets of chromosomes, has long been recognized as a major driver of plant evolution and speciation (Soltis et al., 2009). Naturally occurring polyploids have been identified in a wide range of taxa and recent estimates suggest that almost all extant angiosperms have experienced polyploid events in their evolutionary history (Soltis and Burleigh, 2009). Potential evolutionary/adaptive advantages of being polyploid include increased heterosis, gene redundancy and mutational robustness, and phenotypic plasticity (Comai, 2005; Sattler et al., 2015).

The artificial induction of polyploidy can provide a valuable tool to assist with understanding evolutionary processes and to facilitate plant breeding and improvement programs. Polyploids often possess improved traits, such as thicker, darker-colored leaves; larger, longer-lasting flowers and thicker petals; enhanced vigor; improved tolerances to environmental stresses, pests and pathogens; increased metabolite production and may restore fertility in sterile wide hybrids (Kehr, 1996; Comai, 2005; Ranney, 2006; Banyai et al., 2009). However, incorporating polyploids into plant breeding programs often necessitates the induction of new polyploids.

*In vitro* chromosome doubling has predominantly been induced using the antimitotic agent colchicine. However, the herbicides oryzalin and trifluralin, are often preferred due to their reduced toxicity, higher affinity to plant tubulins, and effectiveness at lower concentrations. The success of *in vitro* chromosome doubling protocols is dependent upon the effectiveness of antimitotic agents to temporarily arrest cell division (cytokinesis) in actively growing tissue. While the length of exposure and concentrations of antimitotic agents is critical for chromosome doubling, several other factors such as tissue types, methods of application, culture conditions and species differences may influence the efficacy of *in vitro* chromosome doubling. Species-specific *in vitro* chromosome doubling protocols for diverse and valuable taxa have been widely reported (Table 1). In this review we will explore mitotic inhibitors and examine factors that impact *in vitro* polyploid manipulation and provide possible areas for further research.

**TABLE 1:** Reported *in vitro* polyploid induction of diverse crops utilizing varied tissues, antimitotic agents and concentrations, and exposure times.

Family	Species	Tissue	Agent	Concentration	Exposure	References
*Actinidiaceae*	*Actinidia chinensis*	Organogenesis from petioles	Colchicine	1.25–2.5 mM	4 h	Wu et al., 2011
*Amaryllidaceae*	*Clivia miniate*	Immature embryos	Colchicine	10–50 mM	10–30 days	Wang and Lei, 2012
	*Allium cepa*	Shoot apices	Colchicine Oryzalin	2.5 mM 50 μM	24 h 24 h	Geoffriau et al., 1997
*Apiaceae*	*Centella asiatica*	Shoot apices	Colchicine	1.25–5 mM	12–24 h	Kaensaksiri et al., 2011
	*Trachyspermum ammi*	Seeds	Colchicine	0.06–1.25 mM	6–48 h	Noori et al., 2017
*Araceae*	*Spathiphyllum wallsii*	Somatic embryos	Oryzalin Trifluralin Colchicine	10 μM 10 μM 100 μM	16 h	Eeckhaut et al., 2004
	*Zantedeschia* sp.	Shoot apices	Colchicine	1.25 mM	1–4 days	Cohen and Yao, 1996
*Asparagaceae*	*Ophiopogon planiscapus*	Embryogenic callus	Oryzalin	7.5 μM	3–9 days	Gillooly et al., 2015
*Asteraceae*	*Echinacea purpurea*	Organogenesis from petioles	Colchicine	300 mM	28 days	Nilanthi et al., 2009
	*Rudbeckia hirta*	Shoot apices	Oryzalin	15 μM	3–5 days	Touchell personal communication
	*Rudbeckia subtomentosa*	Shoot apices	Oryzalin	15–60 μM	3–5 days	Palmer et al., 2008
	*Rudbeckia maxima*	Shoot apices	Oryzalin	60 μM	3 days	Palmer et al., 2008
	*Gerbera jamesonii*	Shoots	Colchicine	2.5–12.5 mM	2–8 h	Gantait et al., 2011
	*Smallanthus sonchifolius*	Nodal segments	Colchicine Oryzalin	3 mM 20–25 μM	24 h 24–48 h	Viehmannová et al., 2009
	*Artemisia annua*	Organogenesis from leaves	Colchicine	25 mM	24 h	Banyai et al., 2009
*Balsaminaceae*	*Impatiens walleriana*	Shoot apices	Oryzalin	15–60 μM	12–48 h	Ghanbari et al., 2019
*Bixaxeae*	*Bixa orellana*	Hypocotyl segments	Oryzalin	15 μM	15 days	de Carvalho et al., 2005
*Brassicaceae*	*Raphanus sativus* x *Brassica oleracea*	Shoots	Amiprophos-methyl	10–30 μM	24 h	Niimi et al., 2015
	*Brassica oleraceae* var. *capitate*	Root cultures	Colchicine	5–10 mM	3–12 h	Yuan et al., 2015
	*Brassica oleraceae* var. *italica*	Root cultures	Colchicine	1.25 mM	6–12 h	Yuan et al., 2015
*Cannabaceae*	*Humulus lupulus*	Shoot apices	Colchicine	0.25–2.5 mM	24–72 h	Trojal-Golush and Skomra, 2013
	*Cannabis sativa*	Shoot apices	Oryzalin	20–60 μM	24 h	Parsons et al., 2019
*Caryophyllaceae*	*Lychnis senno*	Nodal segments	Colchicine	0.25–1.25 μM	3 days	Chen et al., 2006
	*Dianthus caryophyllus*	Nodal segments	APM	32.9 μM	24 h	Nimura et al., 2006
*Cucurbitaceae*	*Citrullus lanatus*	Hypocotyl segments	Colchicine	0.25 mM	4 days	Raza et al., 2003
	*Citrullus lanatus*	Shoot apices	Colchicine Oryzalin Ethalfluralin Butralin Dinitramine	1.5–2.5 mM 25–100 μM 25–100 μM 25–100 μM 25–100 μM	3–9 days 3–9 days 3–9 days 3–9 days 3–9 days	Nasr et al., 2004
	*Cucumis sativus*	Nodal segments/shoot apices	Colchicine Oryzalin Trifluralin	0.6–3.75 mM 15–433 μM 15–450 μM	18–36 h 18–36 h 18–36 h	Ebrahimzadeh et al., 2018
*Ericaceae*	*Rhododendron* ‘Frangrantissimum Improved’	Organogenic callus	Oryzalin	7.5	14 days	Hebert et al., 2010
	*Rhododendron*	Seedlings	Oryzalin Trifluralin	0.3 mM 0.3 mM	3 days 3 days	Eeckhaut et al., 2002
	*Rhododendron*	Shoots	Oryzalin	30 μM	24 h	Väinölä, 2000
*Fabaceae*	*Cercis glabra*	Shoot apices	Oryzalin	150 μM	12–96 h	Nadler et al., 2012
*Haemodoraceae*	*Anigozanthos* sp.	Axillary buds	Colchicine	2.5 mM	7 days	Griesbach, 1990
*Hydrangea*	*Hydrangea macrophylla*	Apical shoot	Oryzalin	15–30 μM	3–5 days	Touchell personal communication
	*Hydrangea arborescence*	Apical shoot	Oryzalin	15–30 μM	3–5 days	Touchell personal communication
*Hypericaceae*	*Hypericum* sp.	Organogenic callus	Oryzalin	30 μM	6 days	Meyer et al., 2009
*Iridaceae*	*Crocosmia aurea*	Seed	Colchicine	0.25–25 μM	12 h–3 days	Hannweg et al., 2013
	*Watsonia lepida*	Hypocotyl segments	Oryzalin	25–250 μM	1–3 days	Ascough et al., 2008
*Lamiaceae*	*Thymus persicus*	Shoot apices	Colchicine	0.75–1.25 mM	12–48 h	Tavan et al., 2015
	*Tetradenia riparia*	Seed	Colchicine	0.025–0.25 mM	12–72 h	Hannweg et al., 2016b
	*Plectranthus esculentus*	Nodal segments	Colchicine	0.025250 mM	12–72 h	Hannweg et al., 2016a
*Liliaceae*	*Tulipa gesneriana*	Flower stems	Oryzalin	1.44–28.8 μM	1–14 days	Chauvin et al., 2005
	*Lilium hybrid*	Bulb segments	Oryzalin	30–200 μM	2–6 h	Chandanie et al., 2011
	*Linum album*	Nodal segments	Colchicine	1.25–5 mM	24–96 h	Javadian et al., 2017
*ıLythraceae*	*Lagerstroemia indica*	Nodal segments	Colchicine	0.25–0.75 mM	10 days	Zhang et al., 2010
*Oleaceae*	*Syringa* sp.	Nodal segments	Colchicine	0.05–0.25 mM	1–2 days	Rose et al., 2000
*Orchidaceae*	*Bletilla striata*	Protocorms	Colchicine	1.25–5 mM	12–48 h	Pan-pan et al., 2018
	*Dendrobium chrysotoxum*	Protocorms	Colchicine	1.0 mM	24 h	Artichart, 2013
*Passifloroideae*	*Passiflora edulis*	Hypocotyl segments	Colchicine Oryzalin	0.025–1.25 mM 5–30 μM	15 days 15 days	Rêgo et al., 2011
*Plantaginaceae*	*Hebe* ‘Oratia Beauty’	Nodal segments	Colchicine Oryzalin	0.5–1.0 mM 11.5–289 μM	48 h 48 h	Gallone et al., 2014
*Plumbaginoidaceae*	*Plumbago auriculata*	Shoot apices	Pendimethalin	800 μM	7 days	Jiang et al., 2020
*Poaceae*	*Miscanthus sinensis*	Shoots	Colchicine Oryzalin	313 μM 5–15 μM	18 h 4–7 days	Petersen et al., 2002, 2003
*Poaceae*	*Miscanthus* x *giganteus*	Shoots	Oryzalin	15 μM	3–5 days	Touchell and Ranney, 2012
	*Panicum virgatum*	Embryogenic callus	Colchicine	1 mM	13 days	Yang et al., 2014
	*Triticum aestivum*	Microspore culture	Colchicine	3 mM	24–48 h	Hansen and Andersen, 1998
*Polemoniaceae*	*Phlox subulata*	Shoot apices	Colchicine	0.125–1.0 mM	10–30 days	Zhang et al., 2008
*Ranunculaceae*	*Ranunculus asiaticus*	Shoots	Colchicine Oryzalin Trifluralin	100–200 μM 0.5–3.0 μM 2.0 μM	16–24 h 6–10 weeks 6–10 weeks	Dhooghe et al., 2009a
	*Helleborus niger*	Shoots	Oryzalin Trifluralin	3 μM 3–10 μM		Dhooghe et al., 2009b
	*Helleborus x nigercors*	Shoots	Oryzalin Trifluralin	3 μM 3–10 μM		Dhooghe et al., 2009b
*Rhamnaceae*	*Ziziphus jijuba*	Shoot apices	Colchicine	1.25–2.5	24–72 h	Gu et al., 2005
*Rosaceae*	*Rosa* ‘Therese Bugnet’	Shoot apices/nodal segments	Oryzalin	5–15 μM	14–28 days	Kermani et al., 2003
	*Rosa rugosa*	Nodal segments	Oryzalin	5 μM	12 h	Allum et al., 2007
	*Rosa hybrida*	Nodal segments	Oryzalin Trifluralin APM	6–24 μM 6–24 μM 6–24 μM	12–48 h	Khosravi et al., 2008
	*Rosa persica*	Nodal segments	Trifluralin APM	6–24 μM 6–24 μM	12–48 h	Khosravi et al., 2008
	*Chaenomeles japonica*	Nodal segments	Oryzalin Colchicine	10–50 μM 0.25–38 mM	1–2 days	Stanys et al., 2006
	*Prunus laurocerasus*	Shoots	Oryzalin	150 μM	1–2 days	Contreras and Meneghelli, 2016
	*Malus* x *domestica*	Axillary buds	Colchicine	10 mM	2 days	Hias et al., 2017
	*Pyrus pyriflora*	Shoots	Colchicine	0.25 mM	1–8 days	Kadota and Niimi, 2002
	*Pyrus communis*	Organogenesis from leaf	Colchicine	1 mM	24–72 h	Sun et al., 2009
*Salicaceae*	*Populus* sp.	Organogenesis from leaves	Colchicine	50–100 μM	2–4 days	Xu et al., 2016
	*Populus hopeiensis*	Organogensis from leaves	Colchicine	100 μM	96 h	Wu et al., 2020
*Sapindaceae*	*Acer platanoides*	Nodal segments	Oryzalin	15 μM	3 days	Lattier et al., 2013
*Scrophulariaceae*	*Buddleja* sp.	Nodal segments	Oryzalin	3–7 μM	1–3 days	Dunn and Lindstrom, 2007
*Solanaceae*	*Petunia axillaris*	Leaves, organogenesis	Colchicine	0.2 mg	15 days	Regalado et al., 2017
*Vitaceae*	*Vitis* sp.	Shoots	Colchicine	1.25 mM	24–48 h	Notsuka et al., 2000
*Zingiberaceae*	*Hedychium muluense*	Embryogenic callus	Colchicine Oryzalin	2.5 mM 20–120 μM	1–3 days 1–3 days	Sakhanokho et al., 2009

## History

Artificial induction of polyploids in plants was first reported in the late 1930s with Blakeslee and Avery (1937) demonstrating the use of colchicine for chromosome doubling of several species. Numerous studies investigating colchicine for ploidy manipulation soon followed this initial report (see Dunham and Banta, 1940). The interest in polyploidy grew rapidly, and in 1941, The American Naturalist published the ‘Symposium on theoretical and practical aspects of polyploidy in crop plants’ (The American Naturalist, 1941). In that issue, Emsweller and Ruttle (1941) first discussed the value of induced polyploidy for the improvement of ornamental plants. Since these early studies chromosome doubling has become an integral component of breeding programs for many economically important crops.

Advancements in plant tissue culture in the 1960s provided new opportunities for developing polyploids. Murashige and Nakano (1966) isolated tetraploid cells from the pith of diploid tobacco plants and used *in vitro* culture to stabilize and produce tetraploid plants. Soon after, Heinz and Mee (1970) reported the use of colchicine to induce polyploid sugarcane cell suspensions. Hussey and Hypher (1978) recovered tetraploid sugar beets by treating *in vitro* grown plantlets with colchicine. The past two decades have seen a significant increase in the use of *in vitro* polyploid induction. This increase may be attributed, in part, to the development and proliferation of tissue culture protocols for a diverse range of taxa.

## Antimitotic Agents

*In vitro* polyploid manipulation is dependent upon disrupting the cell cycle to prevent polar migration of chromosomes during anaphase. Chemicals ranging from caffeine (Thomas et al., 1997) and nitrous oxide (Taylor et al., 1976) to antimicrotubule herbicides have all been shown to induce polyploidy. However, several antimicrotuble compounds, such as colchicine and oryzalin, have been predominantly used for successful *in vitro* polyploid induction (Table 2).

**TABLE 2:** Mitotic inhibitors that are used or have potential to interfere with the cell cycle to induce polyploids.

Mitotic inhibitor	Mode of action	Application
**Miscellaneous**		
Colchicine	Destabilizes β-tubulin	Seeds, shoots, see Table 1
Taxol	Stabilizes β-tubulin	Not reported for plant polyploidy
Nitrous oxide	Possible interacts with α-tubulin	Seeds Taylor et al. (1976)
**Dinitroanilines**		
Oryzalin	Destabilizes α-tubulin	Seeds, nodal segments, shoots, callus, see Table 1 for examples
Trifluralin	Destabilizes α-tubulin	Nodal segments, shoots, callus, see Table 1 for examples
Pendimethalin	Destabilizes α-tubulin	Limited use, Micro-shoots of *Nepta* (Mitrofanova et al., 2003)
Ethalfluralin	Destabilizes α-tubulin	Limited use, Micro-shoots of *Nepta* (Mitrofanova et al., 2003)
**Benzamides**		
Propyzamide	Destabilizes α-tubulin	Nodal segments of *Vitis davidii* (Cai et al., 2016)
*Phosphorothioates*		
Amiprophos-methyl (APM)	Destabilizes α-tubulin, same binding site as oryzalin	Nodal segments (Nimura et al., 2006)
**Cyanoacrylates**		
Ethyl (2Z)-3-amino-2-cyano-4-ethylhex-2-enoate (CA1)	Destabilizes α-tubulin, same binding site as oryzalin	No reports for plant polyploid induction
**Carbamates**		
Propham	Disrupt and fragment spindle poles	No reports for plant polyploid induction
**Proteasome inhibitors**		
Lactacystin	Interfere with regulatory proteins that govern metaphase, anaphase and cytokinesis transitions	No reports for plant polyploid induction
MG132	Same as lactacystin	No reports for plant polyploid induction
**Cancer drugs**		
Reversine	Inhibits anaphase in human breast tissue to form polyploid cells	No reports for plant polyploid induction

Colchicine [*N*-5,6,7,9-tetrahydro-1,2,3,10-tetra-methoxy-9-oxobenzo(a)heptalen-7-yl] acetamide is perhaps the most commonly used mitotic inhibitor and has been used to recover polyploids in a wide range of species (Table 1). Colchicine is extracted from the bulbs of autumn crocus (*Colchicum autumnale*) and is widely used as a medication to treat gout and other inflammatory diseases. As an antimitotic agent, colchicine disrupts the cell cycle beginning at metaphase where it destabilizes microtubules by binding to the β–tubulin subunit to form a colchicine-tubulin complex. As such, colchicine prevents microtubule polymerization, without influencing depolymerization, resulting in degradation of microtubules (Leung et al., 2015). For *in vitro* chromosome doubling, colchicine has advantages that it is soluble in aqueous solutions, heat-stable, and can be autoclaved and easily applied to plant tissues. However, colchicine has high binding affinity for animal microtubules and is potentially toxic to humans (Morejohn et al., 1984). In contrast, colchicine has relatively low binding affinity to plant microtubules which requires it to be used in high concentrations to maintain effectiveness.

Collectively, certain herbicides provide viable alternatives to colchicine for *in vitro* ploidy manipulation. It is estimated that approximately 25% of herbicides act by affecting mitosis (Vaughn and Lehnen, 1991). Herbicides consist of several different chemical classes with antimitotic activity, including dinitroanilines (oryzalin, trifluralin, pendamethalin) (Morejohn et al., 1987; Hugdahl and Morejohn, 1993), phosphorothioamidates (amipro-phos-methyl) (Murthy et al., 1994), benzamides (propyzamide) (Bartels and Hilton, 1973), cyanoacrylates (ethyl (2Z)-3-amino-2-cyano-4-ethylhex-2-enoate) (Tresch et al., 2005), and carbonates (chlorpropham, propham).

Dinitroanilines are the most common class of herbicides used for *in vitro* ploidy manipulation. Dinitroanilines have shown to have high binding affinity to plant tubulins at low concentrations while showing little binding affinity with animal tubulins (Morejohn et al., 1987; Hugdahl and Morejohn, 1993). This group of compounds works similarly to colchicine to disrupt mitosis in metaphase. Dinitroanilines bind to α-tubulin to form a tubulin-dinitroaniline complex to prevent microtubule polymerization.

The dinitroanilines contain numerous compounds that can be divided into symmetric (e.g., oryzalin, trifluralin) and non-symmetric (e.g., pendimethalin) compounds that differentially interact with α-tubulin (see Ma et al., 2010). In a comprehensive study evaluating the effect of 12 different dinitroanilines on the unicellular parasite, *Toxoplasma gondii*, expressing oryzalin sensitive wild-type and α-tubulin mutants conferring oryzalin resistance, Ma et al. (2010) found that non-symmetric compounds dinitramine and pendimethalin demonstrated increased inhibition. Similarly, several trifluralin analogs showed increased binding efficiencies to α-tubulin of the unicellular organism *Trypanosoma brucei* (Giles et al., 2009). These studies have suggested that small species-specific differences in the properties of α-tubulin binding sites may influence interaction with functional groups of different dinitroanilines.

For plant species, studies have shown that mutations to α-tubulin binding sites may alter binding affinities and confer resistance to the dinitroanilines, specifically oryzalin and trfluralin (Anthony and Hussey, 1999; Chu et al., 2018). This may have significance for *in vitro* ploidy manipulation, as studies have been primarily isolated to oryzalin and trifluralin (Table 1), with only limited reports of alternative dinitroanilines such as pendamethalin (Ren et al., 2018), dinitramine (Nasr et al., 2004), ethylfluralin (Mitrofanova et al., 2003), and butralin (Nasr et al., 2004). With the structural diversity in dinitroanilines, different compounds may provide higher efficacy for recovering polyploids in recalcitrant species.

The phosphoric amides are another group of herbicides with antimitotic activity, of which amiprophos-methyl (APM) has been used for *in vitro* chromosome doubling (Khosravi et al., 2008). Amiprophos-methyl has shown high affinity for tobacco α-tubulin and may target the same binding sites as oryzalin (Murthy et al., 1994). An advantage of APM is that it has increased solubility in water compared to dinitroanilines, thus reducing the use of additional solvents. Similarly, the benzamides, particularly propyzamide have shown potential for *in vitro* chromosome doubling (Cai et al., 2016). Propyzamide also targets the same binding sites as oryzalin (Bartels and Hilton, 1973). Cyanoacrylates are another class of antimitotic agents that have the same mechanisms as dinitroanilines (Tresch et al., 2005). Similar to APM and propyzamide, the cyanoacrylates, ethyl (2Z)-3-amino-2-cyano-4-ethylhex-2-enoate (CA1) and CA2 bind to α-tubulin at the same sites as oryzalin (Tresch et al., 2005). However, they have yet to be used for *in vitro* polyploid induction.

Nitrous oxide has also been reported to induce polyploids (Taylor et al., 1976). The mode of action has remained unclear. However, Kitamura et al. (2009) suggested that nitrous oxide may induce polyploidy by inhibiting microtubule polymerization. It is likely that nitrous oxide interacts with tyrosine to form nitrotyrosine (Neill et al., 2003). Nitrotyrosine may replace tyrosine in α-tubulin and influence polymerization (Blume et al., 2013; Lipka and Müller, 2014). Lipka and Müller (2014) found that in *Arabidopsis thaliana* nitrotyrosine alone inhibited microtubule polymerization, but reduced sensitivity to oryzalin due to changes in α-tubulin binding sites. However, Jovanović et al. (2010) demonstrated that nitrotyrosine increased sensitivity of *Nicotiana tabacum* L. cell cultures to oryzalin, suggesting nitrotyrosine could provide addition antimicrotubule qualities.

In contrast to antimicrotubule agents, the carbamate herbicides, such as propham or chlorpropham, act to disrupt mitosis without influencing the polymerization or destabilization of microtubules. Rather, carbamates act to disrupt and fragment spindle poles throughout the cell resulting in a multipolar, rather than bipolar, migration of chromosomes (Vaughn and Lehnen, 1991). As such, it is unlikely that carbamates will be effective in the development of polyploids.

There are no reports of the use of proteasome inhibitors being utilized for vitro ploidy manipulation. However, proteasome inhibitors such as lactacystin and MG132, interfere with key regulatory proteins that govern the metaphase, anaphase and cytokinesis transitions (Planchais et al., 2000). A drawback of proteasome inhibitors is that treatments are not fully reversible (Planchais et al., 2000).

Another group of cell cycle inhibitors, including hydroxyurea and aphididcolin, act to arrest cell cycle at the beginning of S-phase. Following the removal of the inhibitor, cells progress through S, G_2_, and M phase in a synchronized manner (Darzynkiewicz et al., 2011). While these compounds do not directly affect polyploidy, they may have utility as treatments to facilitate synchronizing the cell cycle and maximize the number of cells affected by the antimicrotubule agent thereby reducing cytochimeras.

## *In Vitro* Polyploid Induction – an Overview

The success of *in vitro* polyploid induction is highly integrated with the development of efficient *in vitro* culture protocols. Plant tissue culture systems have often proven difficult for many taxa, especially woody plants and only a limited number of species have successfully been grown in tissue culture. Protocol development often needs to be conducted for each species and often for each clone to optimize regeneration protocols that can be applied for *in vitro* polyploid induction. Nonetheless, successful chromosome doubling has been achieved for a significant number of species representing a diverse range of families and genera (Table 1).

## Key Variables Influencing *In Vitro* Polyploid Induction

### Tissue Type

*In vitro* ploidy manipulation is highly dependent on the availability of successful *in vitro* regeneration systems. Although apical meristems can be treated *in vitro*, regeneration via somatic embryogenesis or shoot organogenesis is highly desirable for polyploid induction treatments. The ability to regenerate an entire plant from a single or only a few cells can improve the development of homogenous polyploid plants and minimizes the possibility of cytochimeras. Organogenic or embryogenic regeneration systems have been used for chromosome doubling for several species (Table 1). For *Echinacea purpurea*, polyloids were regenerated from petioles treated with colchicine (Nilanthi et al., 2009). Similarly, organogenesis from *Populus* sp. leaves treated with oryzalin resulted in polyploids. Sakhanokho et al. (2009) treated embryogenic callus of *Hedychium muluense* with colchicine or oryzalin to develop homogeneous polyploids. Further, *in vitro* regeneration systems are essential for developing dihaploids. Hansen and Andersen (1998) regenerated dihaploids from microspores of *Triticum aestivum* treated with colchicine. In an alternative approach, Yuan et al. (2015) regenerated dihaploids from *in vitro* roots treated with colchicine of haploid *Brassica* sp.

*In vitro* regeneration systems via organogenesis and somatic embryogenesis, however, have only been developed for relatively few species, and this approach may result in greater somaclonal variation due to mutations and epigenetic changes (Bairu et al., 2007). For many crops, the development of *in vitro* regeneration systems provides unique challenges and alternative tissues may need to be considered. As such, nodal segments and shoot apices have been the most widely used tissues for *in vitro* chromosome doubling (Table 1).

To obtain homogenous polyploids using nodal segments and shoot apices, all initial cells within the three histogenic layers of the meristems need to be affected by the antimitotic agent (Dermen, 1953; Klekowski, 2003). If all the initial cells are not affected, mixoploids or cytochimeras may form. Mixoploids have been widely observed in *in vitro* chromosome doubling of a wide range of species, including; *Acer platanoides* (Lattier et al., 2013), *Helleborus* sp. (Dhooghe et al., 2009b), *Hypericum* sp. (Meyer et al., 2009), *Lagerstroemia indica* (Zhang et al., 2010), *Rhododendron* hybrids (Väinölä, 2000; Hebert et al., 2010), *Rosa rugosa* (Allum et al., 2007), *Ranunculus asiaticus* (Dhooghe et al., 2009a), *Tulipa gesneriana* (Chauvin et al., 2005), and *Vitis* sp. (Notsuka et al., 2000; see Table 1).

Mixoploid tissue are often unstable and have a high tendency for diplontic selection and may revert to their original ploidy. Diplontic selection may occur when diploid (or lower ploidy) cells (having less DNA) can replicate and divide faster than neighboring higher ploidy cells (Dermen, 1953; Klekowski, 2003). Over time, the proportion of lower ploidy cells increases resulting in the loss of converted cells. For example, Hussey and Hypher (1978) documented cytochimeral sugar beets after treatment with colchicine and observed that polyploid cells disappeared over subsequent subcultures. Similarly, Lattier et al. (2013) found that for *Acer platanoides*, mixoploid tissues reverted to diploids over a 6-month period.

Another approach is to treat seeds with antimitotic inhibitors prior to establishing *in vitro* cultures. Hannweg et al. (2013) treated *Crocosmia aurea* seed with 0.25 μM colchicine overnight or 25 μM colchicine for 3 days before using introducing them into tissue culture. The highest induction of homogenous tetraploids (29.82%) was achieved with 0.25 μM overnight.

### Selection and Exposure to Antimitotic Agents

Polyploid induction is highly variable between species and cultivars and is dependent upon antimitotic agent, tissue type, and culture conditions (Table 1). While the dinitroaniline herbicides have a high affinity for plant tubulins and, more recently, have been increasingly used for *in vitro* chromosome doubling, colchicine remains a highly effective mitotic agent for many species. Morejohn et al. (1984) showed that binding efficiencies of colchicine to plant tubulins varied substantially between species, and colchicine still remains the most efficient and preferred antimitotic agents for species such as *Populus hopeiensis* (Wu et al., 2020).

Cholchicine is clearly the most used antimitotic agent (Table 1), regardless of the well-documented negative drawbacks (Dhooghe et al., 2009a), and has been used for a diverse species ranging from herbaceous (e.g., *Echinacea purpurea;* Nilanthi et al., 2009) to woody crops (e.g., *Chaenomeles japonica;* Stanys et al., 2006). The wide success of colchicine as an antimitotic inhibitor has led to its continued and regular use in more recent studies, for example *Bletilla striata* (Pan-pan et al., 2018), *Petuna axillaris* (Regalado et al., 2017), and *Populus hopeiensis* (Wu et al., 2020). In comparison, oryzalin has been used successfully to create polyploid lines of woody and semi-woody plants including *Rosa* (Kermani et al., 2003), *Rhododendron* (Hebert et al., 2010), *Chaenomeles* (Stanys et al., 2006), *Hypericum* (Meyer et al., 2009), and *Acer platanoides* (Lattier et al., 2013; Table 1). Further, oryzalin has been shown to be more efficient than colchicine for *in vitro* chromosome doubling for *Chaenomeles japonica* (Stanys et al., 2006), *Rhododendron* sp. (Väinölä, 2000), *Watsonia lepida* (Ascough et al., 2008), and *Ranunculus asiaticus* (Dhooghe et al., 2009a).

The concentration and length of exposure to antimitotic compounds are factors that are consistently investigated. While low levels of exposure are non-effective and high levels are lethal, the interaction between exposure time and concentration is not fully understood. For oryzalin, Meyer et al. (2009) found that the concentration significantly affected survival and ploidy induction of *Hypericum* sp. callus; however, length of exposure had no effect. Similarly, concentration and exposure duration of oryzalin were not significant factors in inducing polyploidy in *Rhododendron* hybrids (Väinölä, 2000). For *Populus hopeiensis* both concentration and exposure time to colchicine were highly significant factors influencing polyploidy. For *Rosa* sp. the relationship between concentration and exposure time to oryzalin seemed to be dependent upon tissue type (Kermani et al., 2003; Allum et al., 2007). Kermani et al. (2003) found reduced duration to oryzalin was required when using 1 mm nodal segments compared to shoot apices. Similarly, Allum et al. (2007) found nodal segment size influenced exposure duration to oryzalin. Allum et al. (2007) suggested that to ensure chromosome doubling, exposure time should be long enough to maximize the number of cells in mitosis and exposure to antimitotic compounds.

Solubility in relation to binding affinity to tubulin is another factor to consider when selecting an antimitotic agent. For example, colchicine is highly soluble in water (>1.5M) and can be readily added to standard culture media, but has a relatively low binding affinity to plant tubulins (Morejohn et al., 1987). In contrast, dinitroanilines are relatively insoluble in aqueous solutions, with oryzalin reaching saturation at < 7.5 μM in water, but often bind to plant tubulins at concentrations below 500 nM (Morejohn et al., 1987). Interestingly, working concentrations of oryzalin commonly used for polyploid induction regularly exceed both binding affinity and solubility concentrations (Table 1). While species differences in oryzalin specificity to tubulins may account for some variability (Ma et al., 2010), the interaction between exogenous oryzalin concentrations and intracellular tubulin remain unclear.

The most common method for the *in vitro* application of antimitotic inhibitors is via treatment in a liquid solution or media, followed by recovering tissues on a regeneration or propagation medium, though, in some cases, the addition of antimitotic agents to solidified media has been successful for developing polyploids. For *Hypericum* sp., polyploids were developed after exposing regenerative callus to oryzalin in a liquid media for 3 to 9 days (Meyer et al., 2009). In contrast, Dhooghe et al. (2009a) used solid media containing oryzalin to induce polyploidy in *Ranunculus asiaticus.*

Commonly, the antimitotic agents are added to media with similar composition to the *in vitro* growth media. It is possible that media components may also interact with antimitotic agents to influence chromosome doubling. For example, the efficiency of dinitroanilines binding to α-tubulin is significantly influenced by pH and sucrose (Morejohn et al., 1987; Hugdahl and Morejohn, 1993). In tubulin binding assays, Hugdahl and Morejohn (1993), showed a threefold increase in oryzalin binding affinity when pH was increased from 6.0 to 7.0. Further, these authors noted that sucrose may alter the interaction between oryzalin and tubulin (Hugdahl and Morejohn, 1993). Sucrose and pH are two key components in plant tissue culture media and need to be considered when developing *in vitro* polyploidy protocols.

Plant growth regulators may also interact with antimitotic agents. Lattier et al. (2013) showed the addition of the cytokinin 6-benzyl amino purine (BA) to medium containing oryzalin increased shoot mortality at higher oryzalin concentrations. They suggested that BA may act to increase cell cycling, producing cells that more quickly transition from metaphase to anaphase where they may be susceptible to oryzalin (Lattier et al., 2013). Considering media components when developing *in vitro* ploidy manipulation protocols may maximize chromosome doubling efficiencies.

## Influence Of Pre and Post-Treatments

In some cases, pre-treatments have been utilized to facilitate synchronizing the cell cycle to maximize the effect of antimitotic agents. Lattier et al. (2013) found for nodal segments of *Acer platanoides*, a 7-day culture on media supplemented with a combination of 4 μM BAP and 1 μM IAA prior to oryzalin treatments, significantly increased the number of stable tetraploids recovered. Similarly, Wu et al. (2020) found leaf blades of *Populus hopeiensis* cultured on 1.78 μM BAP, 0.07 μM TDZ and 0.53 μM IAA for 7 days prior to colchicine treatment significantly increased polyploid induction. In contrast, for *Tulipa gesneriana*, incubating stem disks for 2 weeks on media supplemented with 4 μM BAP, 15 μM 2-iP, and 4 μM NAA did not influence polyploid induction (Chauvin et al., 2005).

Culture conditions following treatment with antimitotic agents have also been shown to influence chromosome doubling. Niimi et al. (2015) found that culturing *Raphanobrassica* hybrids on media containing either silver nitrate (AgNO_3_) or aminoethoxyvinylglycine (AVG) after treatment with the mitotic inhibitor APM increased in tissue survival and polyploids. These compounds inhibit ethylene production and were shown to reduce chlorosis resulting in higher recovery rates of tissues affected by APM.

## Crop Improvement

The effects of whole genome duplication may cause significant genetic changes in gene expression and gene function and may have significant effects for crop breeding and development. The specific effects of polyploidy may vary greatly between species and polyploid induction events.

Morphological changes are regularly reported in response to chromosome doubling. Increased stomatal size has been commonly used to identify chromosome doubling (Stanys et al., 2006; Dhooghe et al., 2010). Traits such as leaf size and thickness (Väinölä, 2000; Dunn and Lindstrom, 2007), inflorescence size and number (Kermani et al., 2003; Allum et al., 2007; Oates et al., 2012) internode length and plant height (Liu et al., 2007; Oates et al., 2012) have all been reported to be influenced by chromosome doubling. Increases in polyploidy have also been linked to an increase in size and biomass. For example, many grasses used as bioenergy feedstocks are polyploids (Lambertini, 2019).

*In vitro* polyploid induction may also facilitate the development of improved, non-invasive, seedless nursery crops. One of the most effective means for developing seedless plants is to create triploids (plants with three sets of chromosomes) by crossing a diploid with artificially induced tetraploid (Ranney, 2006). This approach has been successful for several species including *Miscanthus sinensis* (Rounsaville et al., 2011).

Hybrid sterility, also referred to as chromosomal sterility, often occurs due to improper chromosome pairing during meiosis as a result of structural differences in parental chromosomes (Ranney, 2006; Contreras et al., 2007). In many cases, doubling chromosomes of sterile hybrids, thereby developing allotetraploids, provides a homolog for chromosomes to pair with during meiosis and restores fertility. Doubling chromosomes has been successful in restoring fertility in the wide hybrids *Rhododendron* ‘Fragrant Affinity’ (Contreras et al., 2007), × *Chitalpa tashkentensis* (Olsen et al., 2006), and *Rudbeckia* sp. (Oates et al., 2012). Further, chromsosome doubling restored fertility to the interspecific triploid bioenergy grass, *Miscanthus* × *giganteus* (Touchell and Ranney, 2012).

## Opportunities for the Future

The future development and improvement of efficient, reliable and repeatable *in vitro* ploidy manipulation protocols may consider multiple variables:

(1) The development of effective and efficient regeneration systems. Regeneration through organogenesis and somatic embryogenesis can facilitate reducing cytochimeras and increase the production of homogeneous polyploids. However, regeneration systems have been developed for a small number of crops and in most cases polyploid induction studies have focused on using shoot apices or nodal segments.
(2) Exploring the diversity of mitotic inhibitors. *In vitro* ploidy manipulation studies have focused primarily on a small number of antimitotic agents. Considering the diversity in the structural range of antimicrotubule agents, especially herbicides, there is significant opportunity to further explore their efficacy in *in vitro* ploidy manipulation.
(3) Considering interaction with media components. *In vitro* procedures are often species-specific and each taxa requires unique media compositions. The effect of the interactions of media components on polyploid induction has received little attention. Considering pH, sucrose, and growth regulators may interact with mitotic inhibitors it may be important to integrate *in vitro* protocols with ploidy manipulation.
(4) Refining cultures conditions before and after treatments. Some studies have found pre- and post-treatments beneficial in maximizing survival and homogeneous polyploids. These treatments may act to synchronize the cell cycle and moderate physiological responses to stresses imposed by mitotic inhibitors.

## Author Contributions

DT was the lead author in the preparation of the manuscript. All authors were involved in discussing, formulating, and editing the manuscript.

## Conflict of Interest

The authors declare that the research was conducted in the absence of any commercial or financial relationships that could be construed as a potential conflict of interest.
